# Loss of SMAD1 in acute myeloid leukemia with *KMT2A::AFF1* and *KMT2A::MLLT3* fusion genes

**DOI:** 10.3389/fonc.2024.1481713

**Published:** 2025-01-06

**Authors:** Lisa Dietsche, Kristin Stirm, Veronika Lysenko, Corina Schneidawind, Alexandar Tzankov, Anne Müller, Alexandre P. A. Theocharides

**Affiliations:** ^1^ Department of Medical Oncology and Hematology, University of Zurich and University Hospital Zurich, Zurich, Switzerland; ^2^ Institute of Molecular Cancer Research, University of Zurich, Zurich, Switzerland; ^3^ Pathology, Institute of Medical Genetics and Pathology, University Hospital Basel, University of Basel, Basel, Switzerland

**Keywords:** *KMT2A* rearrangement, acute myeloid leukaemia, SMAD1, TGF-beta signaling, *KMT2A::AFF1*, *KMT2A::MLLT3*

## Abstract

**Introduction:**

*KMT2A*-rearrangements define a subclass of acute leukemias characterized by a distinct gene expression signature linked to the dysfunctional oncogenic fusion proteins arising from various chromosomal translocations involving the *KMT2A* (also known as *MLL1*) gene. Research on the disease pathomechanism in *KMT2A*-rearranged acute leukemias has mainly focused on the upregulation of the stemness-related genes of the *HOX*-family and their co-factor *MEIS1*.

**Results:**

Here we report the *KMT2A::AFF1* and *KMT2A::MLLT3* fusion gene-dependent downregulation of *SMAD1*, a TGF-β signaling axis transcription factor. *SMAD1* expression is lost in the majority of AML patient samples and cell lines containing the two fusion genes *KMT2A::AFF1* and *KMT2A::MLLT3* compared to non-rearranged controls. Loss of *SMAD1* expression is inducible by introducing the respective two *KMT2A* fusion genes into hematopoietic stem and progenitor cells. The loss of SMAD1 correlated with a markedly reduced amount of H3K4me3 levels at the *SMAD1* promoter in tested cells with *KMT2A::AFF1* and *KMT2A::MLLT3*. The expression of *SMAD1* in cells with *KMT2A::AFF1* fusion genes impacted the growth of cells *in vitro* and influenced engraftment of the *KMT2A::AFF1* cell line MV4-11 *in vivo*. In MV4-11 cells *SMAD1* expression caused a downregulation of *HOXA9* and *MEIS1*, which was reinforced by TGF-β stimulation. Moreover, in MV4-11 cells SMAD1 presence sensitized cells for TGF-β mediated G1-arrest.

**Conclusion:**

Overall, our data contributes to the understanding of the role of TGF-β signaling in acute myeloid leukemia with *KMT2A::AFF1* by showing that SMAD1 loss can influence the growth dynamics and contribute to the pathogenic expression of disease driving factors.

## Introduction

Acute leukemias with rearrangement of the *Lysine Methyltransferase 2A* (*KMT2A*, also known as *MLL1*) gene belong to the adverse risk group and are predominantly found in pediatric cases but also in adult acute lymphoblastic and myeloid leukemias (AML) (1, 2). More than a hundred fusion partners are described for translocations involving *KMT2A* (3, 4). Specific fusion genes have been linked to myeloid or lymphoid lineage disease (3).

*KMT2A* fusion genes and their respective fusion proteins are strong oncogenic drivers, which are sufficient to induce leukemic transformation in hematopoietic stem and progenitor cells (HSPCs) by eminently altering gene expression patterns (5–8). The two most important aberrantly expressed genes in *KMT2A*-rearranged leukemias are *HOXA9* and *MEIS1* since their upregulation was shown to be crucial for leukemogenesis (7, 9–11).

KMT2A is part of a multi-protein complex involved in chromatin modification and gene expression regulation. It is a histone-lysine-methyltransferase with a SET domain, which confers H3K4 mono-, di- and trimethylation activity to histone H3 (12–14). H3K4 trimethylation (H3K4me3) is associated with active transcription at promoter regions of genes (15, 16), and KMT2A chromatin occupancy correlates with H3K4me3 presence (15, 17). Other studies showed that H3K4me3 binding by plant homeodomain 3 (PHD3) finger of KMT2A is critical for transcriptional activity at *HOXA9* and *MEIS1* promoters (17). This implicates a role of H3K4me3 in KMT2A-dependent gene regulation. Upregulation of stemness-related and self-renewal-related genes, including *HOXA9*, other *HOX* family members and their co-factor *MEIS1*, constitute a key component of the pathomechanism of *KMT2A-*rearranged leukemogenesis (7, 9, 10, 18, 19).

SMAD1 is part of the transforming growth factor-β (TGF-β) and bone morphogenetic protein (BMP) signaling axes. The TGF-β pathway is crucial in the regulation of cellular processes such as cell growth, differentiation, homeostasis and regeneration throughout tissues. Intriguingly, TGF-β signaling can have opposing effects depending on the cell type and contexts it is active in (20). In hematopoietic cells, it contributes to the regulation of proliferation, differentiation, growth arrest and apoptosis depending on factors such as cell type, differentiation stage and environmental conditions (21, 22). It is widely acknowledged to be a potent negative regulator of hematopoiesis by maintaining hematopoietic stem cell (HSC) quiescence (23–25) and inhibiting the growth of early hematopoietic progenitors (26–28). One of the direct effects of TGF-β is G1 cell cycle arrest mediated by the downregulation of cyclin-dependent kinases (CDKs) and cyclins (29–31) or upregulation of CDK inhibitors such as p15^INK4B^, p21^CIP1^, p27^KIP1^ and p57^KIP2^ (32–39). During normal myeloid differentiation SMAD1 is highly expressed in HSCs, megakaryocyte-erythrocyte progenitors (MEPs) and granulocyte-monocyte progenitors (GMPs) (40). Thereafter, *SMAD1* expression is gradually reduced during myeloid differentiation and consequently low in terminally differentiated cells (41). Interestingly, in the literature SMAD1 has not yet been mentioned in the context of *KMT2A*-rearranged leukemia. However, in diffuse large B-cell lymphoma (DLBCL) and Hodgkin lymphoma aberrant loss of *SMAD1* expression has been involved in the pathogenesis of the disease (42, 43).

Transcriptomic changes in *KMT2A-*rearranged cells involve both upregulation and downregulation of KMT2A targets (12, 44). Here, we describe the loss of the transcription factor SMAD1 in *KMT2A*-rearranged AML with *KMT2A::AFF1* and *KMT2A::MLLT3* fusion genes by analyzing a panel of patient samples and selected cell lines. Moreover, we provide evidence for the potential of SMAD1 to influence leukemic propagation. The purpose of this study was to shed light on the role of TGF-β pathway components, such as SMAD1, in the disease mechanism of acute leukemias.

## Materials and methods

### CRISPR/Cas9 gene editing of *KMT2A* and *SMAD1*

We used a lentiviral delivery system for CRISPR/Cas9-mediated *KMT2A-N* and *SMAD1* gene disruption in MV4-11 and MM6 cells. Lentiviral particles were generated in HEK293T cells, which were cultured in DMEM (Gibco, +10% FBS, +1% penicillin/streptomycin) and polyethylenimine (PEI, Polysciences, MW 25000) was added for plasmid transfection. HEK293T cells were plated in a 10 cm dish and 4 μg of the respective plasmid of interest, 2 μg of packaging plasmid psPAX2 (Addgene plasmid #12260), and 1 μg of envelope plasmid pCMV-VSV-G (Addgene plasmid #8454) were combined. At 48 hours post-transfection, MV4-11 and MM6 cells were infected with virus particles in the presence of polybrene. Antibiotic selection (puromycin and neomycin (G418)) was performed and selected bulk cultures were used for experiments. To generate the *KMT2A* N-terminal knockout (KO), the lentiCRISPRv2 neo (Addgene plasmid #98292) vector was used. *KMT2A* genomic target sequences (exon 2): sgRNA1: TTGTAGGATGAGCAATTCTT, sgRNA2: TCAGAGTGCGAAGTCCCACA. Sequence of the non-targeting control (NTC) sgRNA: GAACAGTCGCGTTTGCGACT. For the *SMAD1* KO, the pKLV2-U6gRNA-PGK-puro-2A-BFP (Addgene plasmid #67974) vector was used with the sgRNA: TTAGCTCAGTTCCGTAACTT.

### ChIP-qPCR

Four million (Mio) cells were crosslinked (10 minutes (min)) in 1% formaldehyde. Subsequently, cell lysis was performed which was followed by MNase digestion for 15 min and sonication for 3 cycles on ice for 30 seconds (s) each (1/10 per s pulse, 29-31% power) with a 30 s break between cycles. For sonication the Bandelin Sonopuls HD2070 machine was used. Input samples were collected after this step. DNA fragments were incubated with 2.5 ug H3K4me3 antibody (Cat #C15410030, Diagenode) or IgG control (ctrl) and afterward reverse crosslinked and purified. We used the Pierce™ Magnetic ChIP Kit (Thermo Fisher Scientific) according to the manufacturer’s instructions. Primers for the quantitative PCR (qPCR) were generated using Primer-BLAST in the Genome Data Viewer and designed to cover the H3K4me3 peak area. To locate the H3K4me3 peaks at the SMAD1 promoter in human hematopoietic cells (*KMT2A* WT), we used the tracks available at the NCBI Genome Data Viewer (45). Results were analyzed using the % Input method. Adjusted CT Input value (adj. CT IN): *adj. CT IN = CT IN – (log2(Input dilution factor))*. % Input: 100*(2^(adj. CT IN – CT IP)). Primers are listed in **Supplementary Table 2** and antibodies are listed in **Supplementary Table 3**.

### Cell cycle assay

Cells were seeded at a density of 0.5 Mio cells/ml in a 12-well plate with three technical replicates for each condition. TGF-β was used at a concentration of 2 ng/ml. The cell cycle phase was measured after 48 hours (h). Cells were stained in CytoPhase™ Violet (BioLegend Cat #425701) at a 1:250 ratio in medium and incubated for 1.5 h at 37°C in the incubator. Samples were analyzed using a BD LSRFortessa™ flow cytometer and data was analyzed with the FlowJo software package (TreeStar).

### *In vivo* xenotransplantation and tissue processing

For the orthotopic xenotransplantation model, female *NOD.Cg-PrkdcscidIl2rgtm1Wjl/SzJ* (NSG) mice at 6-8 weeks of age were intravenously injected into the tail vein with 1x10^7^ cells SMAD1 overexpressing green fluorescent protein (GFP) positive (SMAD1 OE) or empty vector control red fluorescent protein (RFP) positive (RFP ctrl) MV4-11 or MM6 cells in 100 µl PBS. When the first animals showed disease symptoms (rough fur, low activity, paleness), the study was terminated (30-31 days). Spleen cells and bone marrow isolated from the tibia and femur were harvested. Cells were treated with ACK red blood cell lysis buffer pH 7.2–7.4 (150 mM NH4Cl, 10 mM KHCO3, 0.1 mM Na2EDTA) and strained with a 40 μM cell strainer. Engraftment of MV4-11 cells in the spleen and the bone marrow was analyzed by flow cytometry. All animal studies were reviewed and approved by the Zurich Cantonal Veterinary Office (license 132/2019 and their amendments to A.M., license 035/2023 to A.T.).

### Statistical analysis

We performed all statistical analyses on GraphPad Prism 9 (Version 9.5.1). For normally distributed data and comparisons between two groups, we used an unpaired Student’s t-test. For not normally distributed data and comparisons between two groups, we used Mann-Whitney test, also if data in only one of the groups was not normally distributed. For normally distributed data and comparisons between more than two groups we used a one-way ANOVA with Tuckey’s multiple comparisons test. For not normally distributed data and comparisons between more than two groups, we used the Kruskall-Wallis test together with Dunn’s multiple comparisons test, also if data in only one of the groups was not normally distributed. To test for correlation of two variables, we used the Spearman rank order correlation test. P-value summary: ns not significant p>0.05, *p < 0.05, **p < 0.005, ***p < 0.0005, ****p < 0.0001. Error bars represent mean ± standard deviation.

## Results

### *SMAD1* expression is downregulated in AML with *KMT2A* rearrangement

**Figure 1:**
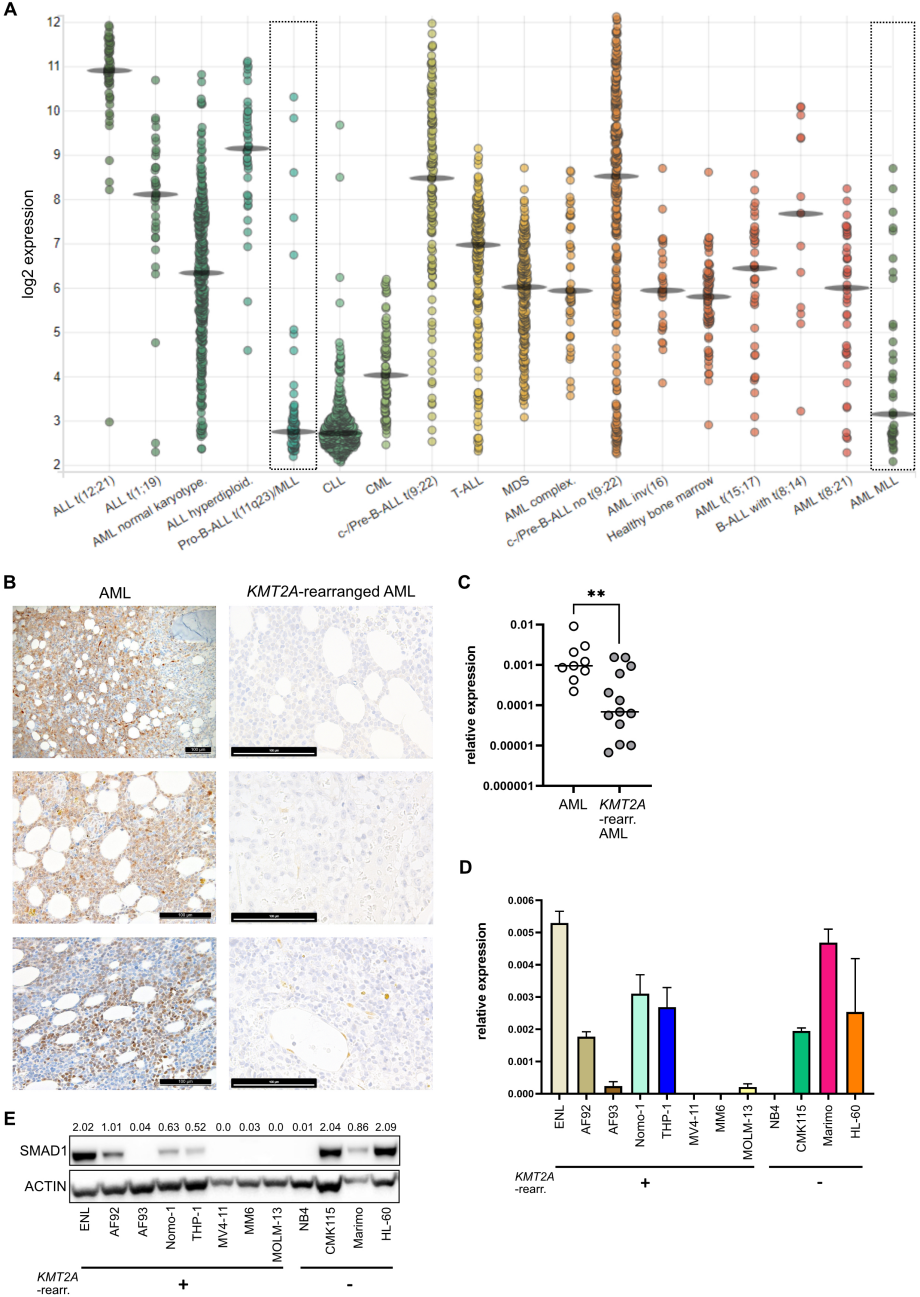
*SMAD1* expression is decreased in *KMT2A::MLLT3* and *KMT2A::AFF1* containing AML patient samples and cell lines. **(A)** *SMAD1* expression in hematologic cancers and healthy bone marrow, as determined by microarray data available on BloodSpot (41). **(B)** Immunohistochemical staining of SMAD1 in bone marrow sections of three representative AML patients without *KMT2A* rearrangement and three representative *KMT2A*-rearranged AML patients (all with *KMT2A::MLLT3)*, scale bar represents 100 µM. **(C)** qPCR relative expression of *SMAD1* in AML with and without *KMT2A* rearrangement (eleven *KMT2A::MLLT3*, one *KMT2A::AFF1*, one *KMT2A::AFDN*) patients (each dot represents one patient sample, each 3 technical replicates). **(D)** RT-qPCR relative expression of *SMAD1* in AML cell lines with and without *KMT2A* rearrangement, 2-5 biological replicates per cell line, each 3 technical replicates. **(E)** Western blot of SMAD1 in AML cell lines with and without *KMT2A* rearrangement. Numbers display densitometry (adjusted lane volume normalized to the respective loading control). Statistics: in **(C)** Mann-Whitney-U test, **p<0.005.

*In silico* analysis of various hematopoietic malignancies showed that, *SMAD1* expression is significantly heterogeneous in the peripheral blood and bone marrow, with particularly low levels observed in chronic lymphocytic leukemia (CLL) and acute leukemia with *KMT2A* rearrangements (**Figure 1A**) (41).

To assess the baseline expression and expressional distribution of SMAD1 in normal bone marrow, we analyzed bone marrow biopsies from four healthy hematopoietic cell donors. Single erythroid precursors within erythrons and HSCs displayed positive SMAD1 staining, while almost all other bone marrow cells remained negative (**Supplementary Figure 1A**). Next, we analyzed bone marrow specimens from AML patients, each six with and without *KMT2A* rearrangement. Four of the six *KMT2A*-rearranged samples contained the *KMT2A::MLLT3* fusion gene, one the *SEPTIN9* fusion gene and one an unknown fusion partner. SMAD1 expression was absent in all four samples with *KMT2A::MLLT3* in comparison to other AML subtypes (**Figure 1B**), confirming the transcriptional data retrieved from the public database BloodSpot. SMAD1 expression in the other two *KMT2A*-rearranged samples was dim but detectable (**Supplementary Figure 3B**). Significantly lower *SMAD1* expression was confirmed by reverse transcriptase quantitative PCR (RT-qPCR) analysis of a different cohort including bone marrow samples from AML patients with and without *KMT2A* rearrangement (**Figure 1C**). We next aimed to identify AML cell lines with *KMT2A* rearrangement and low *SMAD1* expression for mechanistic *in vitro* studies. Additionally, we assessed *SMAD1* expression in human cord blood (CB)-derived cell lines genetically modified to carry *KMT2A* fusion genes *KMT2A::MLLT1* (ELN) and *KMT2A::MLLT3* (AF92 and AF93) (46). Both, RT-qPCR and Western blot analyses revealed that the three *KMT2A*-rearranged cell lines MV4-11 (*KMT2A::AFF1*), MM6 (*KMT2A::MLLT3*) and MOLM-13 (*KMT2A::MLLT3*) and the CB-derived cell line AF93 (*KMT2A::MLLT3*), recapitulate the low expression levels of *SMAD1* observed in AML patients with *KMT2A* rearrangement. Of note, the *KMT2A::MLLT3* containing cell lines Nomo-1 and THP-1 showed an intermediate *SMAD1* expression, not a complete loss. In contrast, the CB-derived cell line with *KMT2A::MLLT1* demonstrated comparably high SMAD1 levels, consistent with findings in primary T-ALL patient cells with *KMT2A::MLLT1* (47) (**Figures 1D, E**). Overall, these findings demonstrate an evident correlation between *KMT2A::AFF1* and *KMT2A::MLLT3* fusion genes and low SMAD1 levels.

### Loss of SMAD1 is linked to the presence of KMT2A fusion proteins

**Figure 2:**
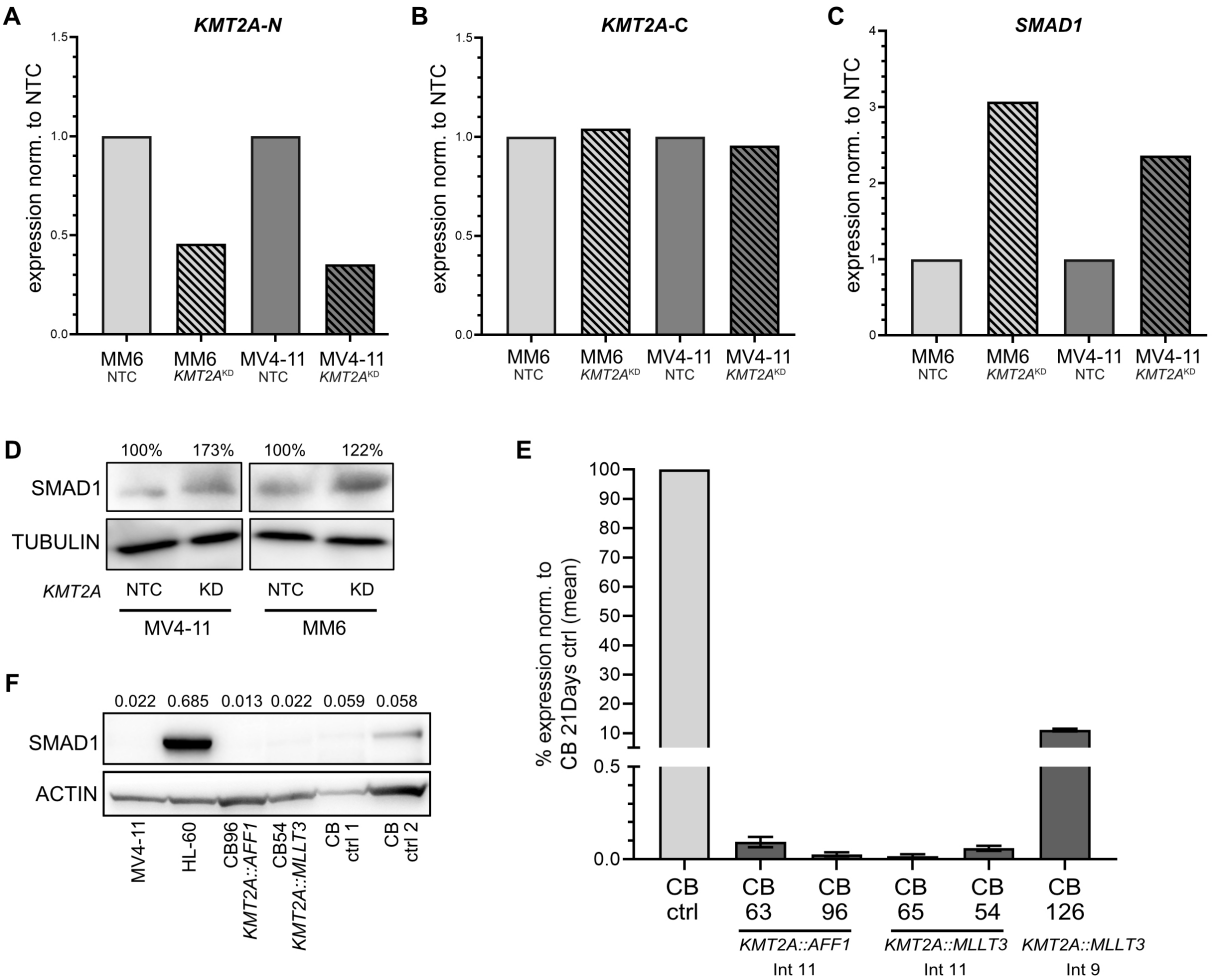
*SMAD1* expression is linked to *KMT2A::MLLT3* and *KMT2A::AFF1* containing AML cell lines and cord blood cells. **(A)** CRISPR/Cas9-mediated *KMT2A*-N KD in MM6 (*KMT2A::MLLT3*) and MV4-11 (*KMT2A::AFF1*) cells. RT-qPCR expression of *KMT2A*-N normalized to NTC **(B)**, expression of *KMT2A*-C normalized to NTC **(C)** and expression of *SMAD1* normalized to NTC. For **(A-C)** (3 technical replicates) **(D)** Western blot of SMAD1 in MV4-11 and MM6 with *KMT2A*-N KD and NTC (long exposure time), percent values are based on densitometry (adjusted lane volume normalized to respective loading control, KD normalized to NTC). **(E)** RT-qPCR expression of *SMAD1* in CB-derived cell lines with *KMT2A::AFF1* and *KMT2A::MLLT3* with different intron breakpoints normalized to cultured CD34+ CB-derived control cells (mean expression of CB ctrl 1 and CB ctrl 2), 3 technical replicates. **(F)** Western blot of SMAD1 in *KMT2A*-rearranged cell lines, CB-derived cell lines with *KMT2A::AFF1* and *KMT2A::MLLT3* and cultured CD34+ CB-derived control cells. Numbers display densitometry (adjusted lane volume normalized to the respective loading control).

To investigate whether *SMAD1* expression correlates with the presence of the KMT2A fusion proteins KMT2A::AFF1 and KMT2A::MLLT3, we next employed CRISPR/Cas9 technology to genetically reduce *KMT2A* fusion gene expression in MM6 and MV4-11 cells. We used a single-guide RNA (sgRNA) designed to target the N-terminal part of the *KMT2A* gene, which is conserved in most *KMT2A* fusion genes (**Figure 2A**; **Supplementary Figure 2A**). Notably, by targeting the N-terminal region of the *KMT2A* gene the wild-type *KMT2A* allele may also be affected. The CRISPR/Cas9 editing led to a 50% reduction of the *KMT2A-*N-terminal mRNA, while the *KMT2A*-C-terminal gene expression remained unaffected, which confirmed the on-target editing effect (**Figure 2B**). We observed a 3-fold increase in *SMAD1* mRNA in MM6 and a 2-fold increase in MV4-11 cells by RT-qPCR analysis (**Figure 2C**). Moreover, the densitometry of the western blots showed that also protein levels of SMAD1 were increased in *KMT2A-N* KD MV4-11 (173%) and MM6 (122%) cells compared to the respective NTC controls (**Figure 2D**). To confirm that *SMAD1* expression levels are directly affected by KMT2A fusion proteins, we examined CB-derived CD34+ cells containing common *KMT2A* fusion genes with different breakpoints that had been engineered by CRISPR/Cas9-mediated gene editing (48). In line with the data generated in cell lines, cells derived from various CB donors showed a strong reduction of *SMAD1* mRNA levels upon introduction of the fusion genes *KMT2A::AFF1* and *KMT2A::MLLT3* (**Figure 2E**). Specifically, *KMT2A::MLLT3* with intron 9 breakpoint caused a reduction to 11%, while *KMT2A::MLLT3* and *KMT2A::AFF1* with an intron 11 breakpoint to 0.01% of *SMAD1* mRNA levels of CD34+ CB cells cultured for 21 days. Similar effects were observed at the protein level, where the densitometry showed that *KMT2A::AFF1* and *KMT2A::MLLT3* containing cells have 2-3 fold lower SMAD1 protein levels compared to CD34+ CB control cells (**Figure 2F**). These results infer a direct role of *KMT2A* fusion genes and presumably KMT2A fusion proteins, in the loss of *SMAD1* expression.

### H3K4me3 levels at the *SMAD1* promoter are reduced in AML with *KMT2A::AFF1* and *KMT2A::MLLT3*

**Figure 3:**
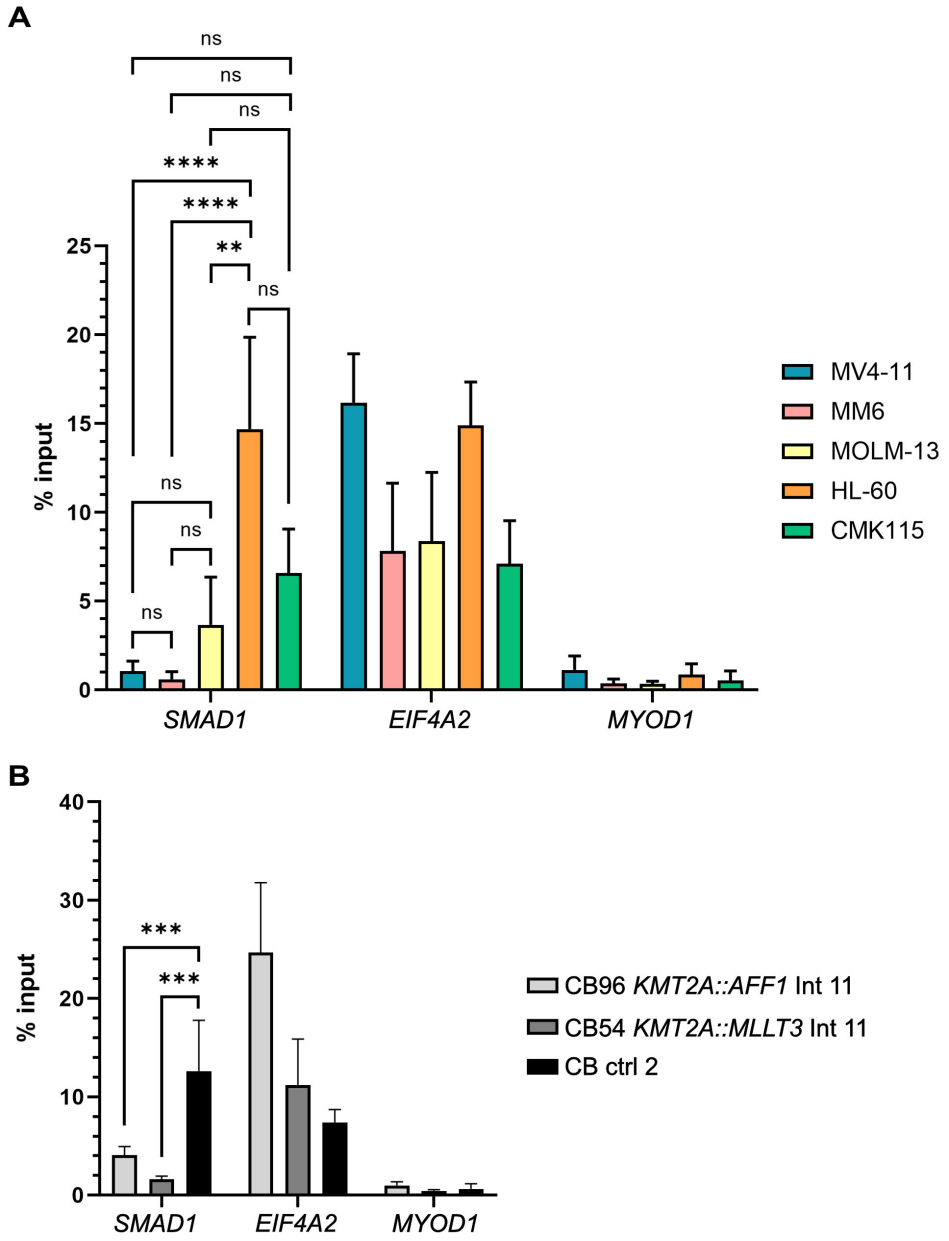
H3K4 trimethylation is reduced at the *SMAD1* locus in *KMT2A::MLLT3* and *KMT2A::AFF1* containing AML cell lines and cord blood cells. **(A)** ChIP-qPCR of H3K4me3 at the *SMAD1*, *EIF4A2* (positive control), *MYOD1* (negative control) promoters in AML cell lines; three biological replicates for all cell lines except CMK115 (two biol. repl.), two biological replicates contain two technical replicates and one biological replicate contains three technical replicates. **(B)** ChIP-qPCR of H3K4me3 at *SMAD1*, *EIF4A2* (positive control), *MYOD1* (negative control) promoters in CB-derived cell lines with *KMT2A::AFF1* and *KMT2A::MLLT3* and cultured CD34+ CB-derived control cells (CB ctrl 2), two biological replicates with once two and once three technical replicates. Statistics: in **(A, B)** one-way ANOVA with Tukey’s multiple comparisons test, ns p>0.05, **p<0.005, ***p<0.0005, ****p<0.0001.

To determine the mechanism of *SMAD1* downregulation in the presence of *KMT2A* fusion genes, we sought to investigate the role of epigenetic alterations. In diffuse large B-cell lymphoma (DLBCL), DNA hypermethylation at the *SMAD1* promoter leads to SMAD1 and subsequent surface receptor sphingosine 1 phosphate receptor 2 (S1PR2) loss, a process that is reversible by treatment with hypomethylating agents (43, 49). However, available Cancer Cell Line Encyclopedia (CCLE) datasets (50) indicate the absence of hypermethylation of the *SMAD1* promoter and reveal no correlation between its methylation and *KMT2A* rearrangement status (**Supplementary Figures 3A, 3B**). Since H3K4 trimethylation belongs to the enzymatic functions of KMT2A and the enzymatic domain is lost in KMT2A fusion proteins we analyzed the H3K4me3 status of the *SMAD1* promoter in *KMT2A* WT and *KMT2A-*rearranged cells using chromatin immunoprecipitation coupled with qPCR (ChIP-qPCR) in AML cell lines. H3K4me3 levels at the *SMAD1* promoter in *KMT2A* WT and SMAD1 positive HL-60 cells were significantly higher than in SMAD1 negative MV4-11, MM6 and MOLM-13 cells (12.4% input versus 1.1%, 0.7% and 3.7% input, respectively, **Figure 3A**). CMK115 cells, which are *KMT2A* WT and SMAD1 positive also showed a similar yet not significant trend of higher H3K4me3 levels at the *SMAD1* promoter compared to the three tested *KMT2A*-rearranged cell lines. Next, we performed the same analysis for the two CRISPR-engineered CB-derived cell lines, CB96 *KMT2A::AFF1* intron 11 (4.0% input) and CB54 *KMT2A::MLLT3* intron 11 (1.6% input), and compared it to control CD34+ CB-derived cells (CB ctrl 2, 12.6% input). Hence, *KMT2A::AFF1* and *KMT2A::MLLT3* containing CB-derived cell lines also showed significantly lower H3K4me3 levels at the *SMAD1* promoter compared to CB-derived CD34+ cells (**Figure 3B**). Considering the role of H3K4 trimethylation in the regulation of gene transcription, these results suggest that reduced H3K4me3 levels at the *SMAD1* promoter in *KMT2A*-rearranged cells could contribute to the loss of *SMAD1* expression.

### *SMAD1* re-expression causes cell cycle arrest and downregulation of *HOXA9* and *MEIS1* expression in MV4-11 cells

**Figure 4:**
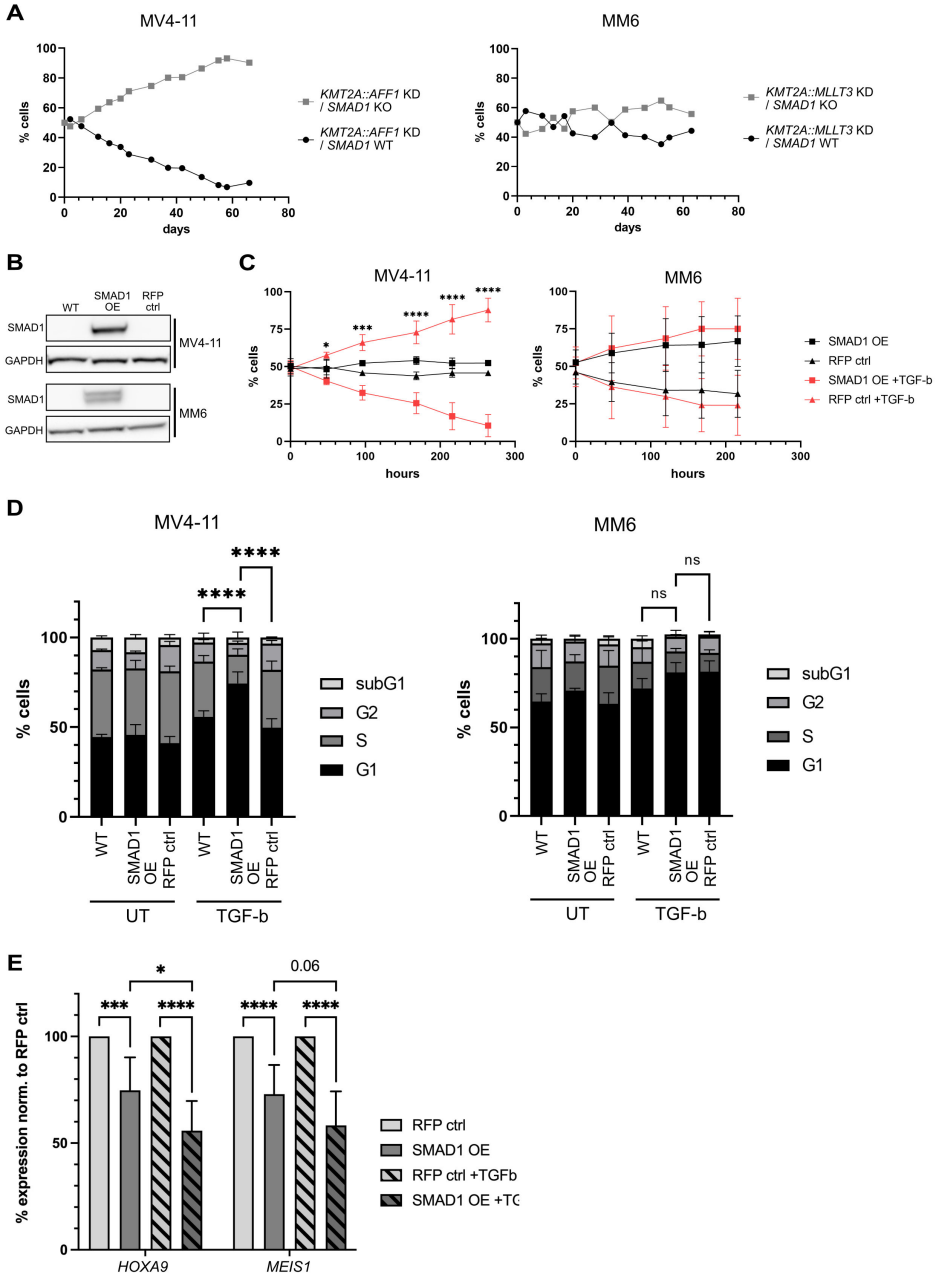
*SMAD1* overexpression in *KMT2A::MLLT3* and *KMT2A::AFF1* containing AML cell lines has diverging effects on growth, cell cycle and expression of *HOXA9* and *MEIS1*. **(A)** Growth competition assay with *KMT2A* KD vs *KMT2A* KD/*SMAD1* KO in MV4-11 and MM6 cells, t=0 ratio 1:1. **(B)** Western blot SMAD1 levels upon *SMAD1* overexpression (OE) in MV4-11 and MM6 cells. **(C)** Growth competition assays of MV4-11 and MM6 cells SMAD1 OE vs RFP control (ctrl) (statistics for MV4-11: comparison between SMAD1 OE +TGF-β and RFP ctrl +TGF-β), two biological replicates. **(D)** Cell cycle assay with MV4-11 and MM6 cells treated as indicated; % of cells in indicated cell cycle phases, 48h treatment, TGF-β 2 ng/ml (three biological replicates for MV4-11 and two biological replicates for MM6 with each three technical replicates of each condition, comparison of G1 phase). **(E)** *HOXA9* and *MEIS1* expression in MV4-11 cells with and without *SMAD1* expression, three biological replicates with three technical replicates each. Statistics: in **(C-E)** unpaired Student’s t-test, ns p>0.05, *p<0.05, ***p<0.0005, ****p<0.0001.

Next, we aimed to investigate the role of SMAD1 in cellular processes influenced by TGF-β such as cell cycle and proliferation in cells with *KMT2A::AFF1* and *KMT2A::MLLT3* fusion genes. To study a potential growth-reducing effect of the SMAD1 rescue (shown in **Figures 2C, D**), we generated a *SMAD1* KO in MV4-11 and MM6 cells with *KMT2A* fusion gene KD. *KMT2A* fusion gene KD cells combined with a non-targeting control are capable of re-expressing *SMAD1*, while *KMT2A* fusion gene KD cells with an additional *SMAD1* KO are not. This allows to attribute differences in functional assays to the re-expression of *SMAD1*. Co-culture of MV4-11 cells containing the two different combinations revealed an outgrowth of *KMT2A::AFF1* KD/*SMAD1* KO cells, the cells that lack *SMAD1* re-expression capacity (**Figure 4A**). However, in MM6 cells no clear outgrowth of cells with *KMT2A::MLLT3* KD/*SMAD1* KO was detected (**Figure 4A**). These results show that re-expression of *SMAD1* upon KD of different *KMT2A* fusion genes in two cell lines can have diverging effects.

To better understand the growth-regulating effects of SMAD1, we overexpressed *SMAD1* (SMAD1 OE) in SMAD1 negative *KMT2A*-rearranged cell lines MV4-11 and MM6, and performed *in vitro* growth competition assays with empty vector control cells (RFP ctrl) (**Figure 4B**). Additionally, we assessed the importance of pathway activation for SMAD1-dependent effects, by adding TGF-β to the co-cultures. In MV4-11 cells, *SMAD1* expression without TGF-β did not affect cell growth. However, in the presence of TGF-β, there was a prominent growth disadvantage of *SMAD1*-expressing cells compared to control cells (**Figure 4C**). In contrast, neither SMAD1 alone nor the addition of TGF-β significantly affected cell growth in MM6 cells (**Figure 4C**). SMAD1 enabled growth reduction in MV4-11 cells, while MM6 cells were insensitive to both SMAD1 and additional TGF-β pathway activation. To further reveal underlying mechanistic reasons for this discrepancy, we performed cell cycle analysis, given the well-known role of TGF-β signaling in cell cycle regulation, especially in hematopoietic cells (26, 51, 52). *SMAD1*-expressing MV4-11 cells displayed a significant G1-arrest of 30% increase upon treatment with TGF-β compared to wild-type (WT) and RFP ctrl cells, which showed a minor response to TGF-β treatment (**Figure 4D**). On the contrary, in MM6 cells, *SMAD1* expression combined with TGF-β treatment did not significantly change the cell cycle phase distribution compared to WT and RFP ctrl cells (**Figure 4D**), consistent with the results of the growth competition assay (**Figure 4C**).

We tested the effect of SMAD1 on *HOXA9* and *MEIS1* expression levels with and without TGF-β. Indeed, SMAD1 significantly decreased *HOXA9* mRNA levels by 25%, which was further decreased by the addition of TGF-β to 44% (**Figure 4E**). *MEIS1* mRNA levels were decreased by 27% upon *SMAD1* expression and TGF-β addition caused a 41% reduction. The expression of both genes was normalized to their expression in RFP control cells with and without TGF-β. Overall, these findings suggest that SMAD1 can play a role in growth regulation by cell cycle control and downregulation of the critical oncogenes *HOXA9* and *MEIS1* in *KMT2A::AFF1* containing MV4-11 cells.

### SMAD1 reduces engraftment of AML cells with *KMT2A::AFF1 in vivo*

**Figure 5:**
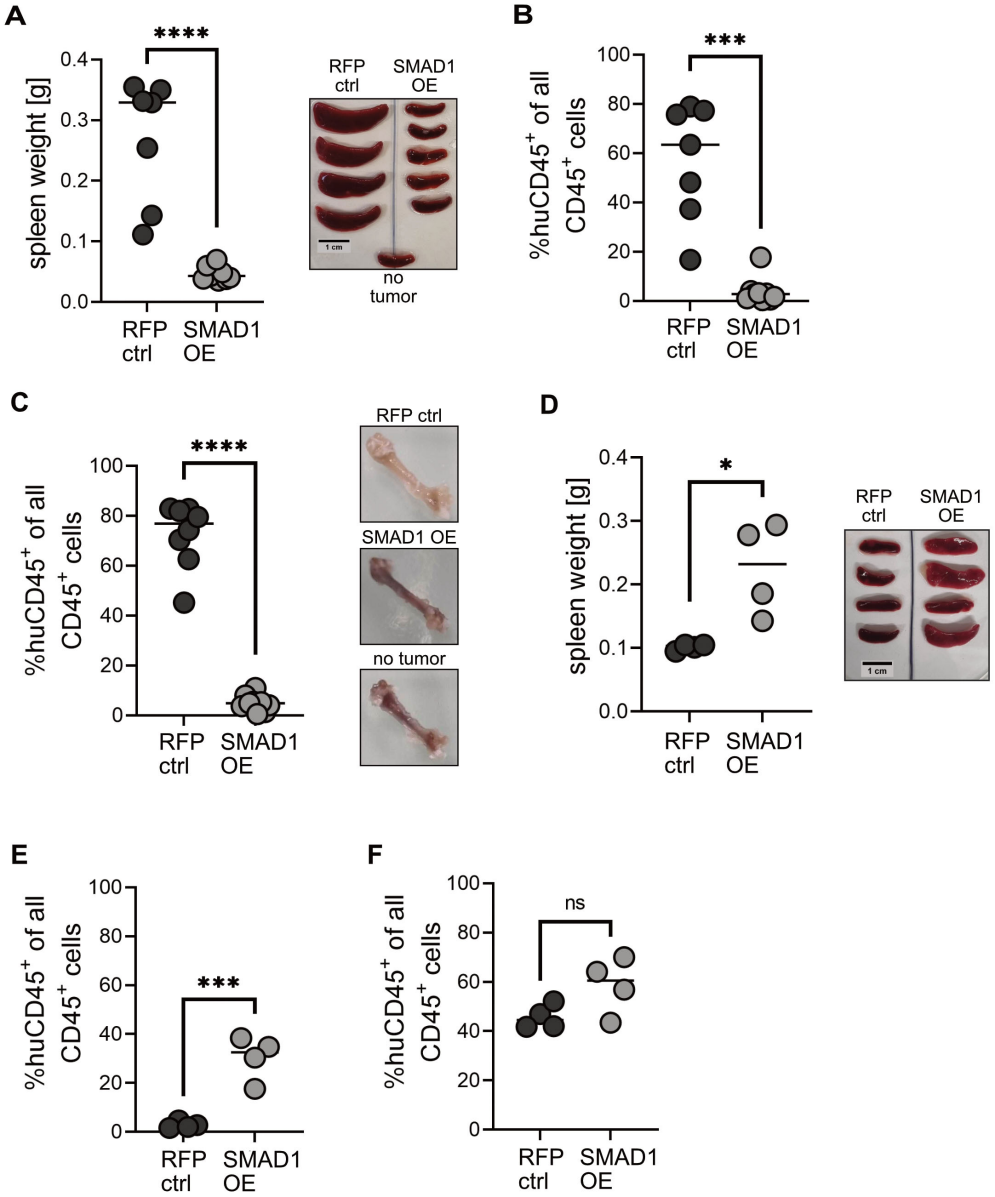
*SMAD1* overexpression in a *KMT2A::AFF1* cell line-derived xenograft model reduces engraftment in the bone marrow and spleen. **(A)** Spleen weight and representative spleen images of NSG mice transplanted with indicated MV4-11 cells. **(B)** Comparison of % human CD45+ cell engraftment in the spleen and **(C)** bone marrow of NSG mice 31 days after orthotopic transplantation (10 Mio cells, i.v. tail vein, mice 6-8 weeks old) of MV4-11 cells with *SMAD1* overexpression (SMAD1 OE) or RFP control (RFP ctrl), with representative femur images. **(D)** Spleen weight and representative spleen images of NSG mice transplanted with indicated MM6 cells. **(E)** Comparison of % human CD45+ cell engraftment in the spleen and **(F)** bone marrow of NSG mice 31 days after orthotopic transplantation (10 Mio cells, i.v. tail vein, mice 6-8 weeks old) of MM6 cells with *SMAD1* overexpression (SMAD1 OE) or RFP control (RFP ctrl). Each dot represents one mouse, in **(A)**, **(C-F)** unpaired Student’s t-test, in **(B)** Mann-Whitney-U test. ns p>0.05, *p>0.05, ***p<0.0005, ****p<0.0001.

To validate the growth-reducing effects of SMAD1 *in vivo*, we tested SMAD1 OE and RFP ctrl MV4-11 cells in a xenotransplantation model, where 10 Mio cells were injected intravenously into the tail vein of NSG (*NOD.Cg-Prkdc^sc^i^d^ Il2rg^tm^1^Wjl^/SzJ*) mice. Four weeks post transplantation, mice were euthanized and the engraftment of human CD45+ cells in the bone marrow and the spleen was determined. We detected a significant reduction in bone marrow and spleen engraftment in mice transplanted with SMAD1 OE cells compared to mice transplanted with RFP ctrl cells (**Figures 5B, C**). This was also reflected by a significantly lower spleen weight in mice transplanted with SMAD1 OE cells (**Figure 5A**).

Conversely, we observed inconsistent engraftment differences in the comparative xenotransplantation with SMAD1 OE and RFP ctrl MM6 cells. SMAD1 OE MM6 cells caused higher spleen weight (**Figure 5D**) and reached higher engraftment levels in the spleen (**Figure 5E**), contrasting with the findings in MV4-11 cells. The engraftment in the bone marrow did not significantly differ between SMAD1 OE and RFP ctrl MM6 cells (**Figure 5F**).

These results support the *in vitro* data. Interestingly, we observed higher overall engraftment of MV4-11 cells in female compared to male NSG mice (**Supplementary Figure 4B**), which was also observed in earlier studies investigating the engraftment of human HSCs (53).

## Discussion

We here provide evidence that *SMAD1* expression is lost or reduced in the majority of AML patient samples and seven AML cell lines, harboring *KMT2A::MLLT3* and *KMT2A::AFF1* fusion genes (three cell lines with reduced and four with absent SMAD1 expression). Notably, one out of four non-rearranged cell lines also showed lack of *SMAD1* expression. We demonstrate that *SMAD1* repression is dependent on the presence of *KMT2A::MLLT3* and *KMT2A::AFF1* fusion genes and that attenuation of the expression of these two fusion genes leads to the re-expression of *SMAD1* in MV4-11 and MM6 cells. Moreover, we confirmed the impact of *KMT2A::MLLT3* and *KMT2A::AFF1* fusion genes on *SMAD1* expression in CB-derived cells. These results confirm previous studies that have shown that *SMAD1* expression can be disturbed by *KMT2A* deficiency (12). In our study this pathobiology was observed in *KMT2A::MLLT3* and *KMT2A::AFF1* containing models and not in other models containing different *KMT2A* fusion genes.

Interestingly, *SMAD1* repression seems to be a recurrent but not a coercive phenomenon in *KMT2A*-rearranged malignancies (41). One limitation of this study is that the assessment of the relationship between *SMAD1* expression and *KMT2A* fusion genes was only performed for the two selected fusion genes *KMT2A::MLLT3* and *KMT2A::AFF1*. Theoretically, there are multiple potential mechanisms for the diverging effects of *KMT2A* fusion genes on *SMAD1* expression such as different interaction partners recruited into the KMT2A fusion protein complex or variable activity of counteractive *SMAD1* regulating pathways.

The loss or disruption of TGF-β pathway components is a pattern observed in other malignancies (54). For example SMAD1 levels are particularly low in DLBCL subtypes (43), but also in Hodgkin lymphoma (42). In T-ALL patient cells, absence of SMAD3 has been reported, correlating with insensitivity to growth-inhibitory effects of TGF-β. While SMAD3 heterozygous or homozygous KO was insufficient to induce T-cell leukemogenesis, it showed growth-promoting effects when combined with homozygous KO of the tumor suppressor *p27^Kip1^* (55). Interestingly, despite its oncogenic potential, inactivating mutations involving TGF-β pathway components are surprisingly rare in hematopoietic malignancies with only a few instances reported, such as TGFβRII mutations in T-ALL (56, 57). Although SMAD4 has been described as a tumor suppressor protecting from the leukemic transformation of HSPCs (58), only very few cases of *SMAD4* mutations in leukemia have been reported (21, 56, 59).

We showed reduced H3K4me3 levels at the *SMAD1* promoter in *KMT2A*-rearranged cell lines, representing a first hint on a potential mechanism for the loss of SMAD1. Yet, at this stage this finding is just an observation without a proof for a causal relationship, which represents an evident limitation of this study. The interaction of KMT2A with H3K4me3 marks was shown to be important for transcription of KMT2A target genes (60, 61). In general H3K4me3 is found at active gene promoters (16), and low H3K4me3 levels are linked to reduced transcriptional output (62). Conversely, although the expression of KMT2A target genes was altered in *KMT2A*-deficient hematopoietic cells, the levels of H3K4 methylation remained stable, indicating that target gene expression and KMT2A fusion protein-driven leukemogenesis are independent of the histone methyltransferase activity of KMT2A (63). This infers that potentially the reduction of H3K4me3 levels at the *SMAD1* promoter is not caused by a lack of KMT2A fusion protein H3K4 trimethylation activity but might stem from the altered activity of other histone methyltransferases. Another study highlighted the importance of H3K4me3, generated by KMT2B (also known as MLL2), for the expression of certain KMT2A::MLLT3 fusion protein target genes and reduced cell viability of *KMT2A*-rearranged cells (64). This suggests that KMT2B is a potential source for H3K4me3 and is crucial for KMT2A WT and KMT2A fusion protein-dependent gene expression. Understanding whether the H3K4 trimethylation activity of KMT2B is disturbed in *KMT2A*-rearranged leukemia, and particularly in context with *KMT2A::AFF1* and *KMT2A::MLLT3* fusion genes, represents an intriguing next chapter in illuminating the pathobiology of *KMT2A*-rearranged leukemia.

TGF-β signaling is a negative regulator in HSPCs (23–28, 55). This study contributes to a deeper understanding of TGF-β signaling and the role of SMAD1, particularly concerning its potential effects on tumor growth in *KMT2A*-rearranged leukemia. *In vitro*, the growth-reducing effect of SMAD1 in MV4-11 cells was only unleashed by the addition of TGF-β. In our xenotransplantation model, we refrained from administering TGF-β to the mice during the study due to the high homology between human and murine TGF-β (65) and since TGF-β is expressed in sufficient amounts in the bone marrow of mice (65, 66). The diminished growth of *SMAD1* expressing MV4-11 cells was confirmed *in vivo*. Loss of TGF-β signaling has been implicated in leukemic progression, due to increased cell cycle induction in HSCs (21). Our findings suggest that the loss of SMAD1 in *KMT2A*-rearranged leukemia with *KMT2A*::*AFF1* promotes tumor growth. The growth-reducing effects were characterized by TGF-β-dependent G1-arrest. However, in the scope of this study we only show this in the *KMT2A*::*AFF1* AML (MV4-11) model, which restricts the impact of our findings and does not allow for extrapolation to other *KMT2A:.AFF1* harboring leukemias.

Further, our data showed a dampening effect of SMAD1 presence combined with TGF-β stimulation on the expression of the two *KMT2A*-rearranged leukemia-driving genes *HOXA9* and *MEIS1*, which has not been described in literature yet. A potential mechanism for this effect, at least for the part of HOXA9, is provided by the findings of Wang et al., which revealed that SMAD1, alongside SMAD4, can bind to HOXA9 upon TGF-β activation, thereby preventing HOXA9-induced gene expression and consequently impeding leukemic transformation (67). Notably, HOXA9 self-regulates its expression via a positive feedback loop and scavenging of HOXA9 by SMAD1 was shown to inhibit this effect (67). If the demonstrated moderate impact on the expression of these two important leukemic drivers in *KMT2A*-rearranged leukemia has a disease modifying effect remains to be confirmed, which will be part of future investigations.

The opposing results of *SMAD1* expression in MM6 cells demonstrate that TGF-β signaling can have diverse outcomes in *KMT2A*-rearranged leukemia, compatible with the versatile nature of TGF-β signaling (20, 68). The effects of the TGF-β signaling pathway and its components are highly context-dependent and hence exhibit a duality in the context of carcinogenesis as well (20, 54, 69). The divergent effects of *SMAD1* expression and TGF-β pathway activation between MV4-11 and MM6 cells may be influenced by factors impacting signaling outcomes, such as post-translational modifications of SMAD1. In mammalian cells, SMAD1 signaling is affected by Mitogen-activated protein kinase (MAPK) and Wingless-related integration site (Wnt) signaling, which phosphorylates its linker region, resulting in reduced nuclear translocation and degradation of SMAD1 respectively (70, 71). Western blot analysis of SMAD1 in MM6 cells showed a second, slightly heavier band than the main band of SMAD1 (**Figure 4B**). This second band could represent a post-translationally modified version of SMAD1, potentially influencing the signal transduction activity of SMAD1. In addition, other factors, such as interfering transcription factors, may influence the outcome of TGF-β pathway activation. In neuroepithelial and glioblastoma cells, the growth inhibitory effects mediated by the interaction of forkhead box (FoxO) transcription factors and SMADs were impeded by FoxG1 and caused reduced expression of the CDK inhibitor *p21^Cip1^* (72). Although the interaction of FoxOs was shown for SMAD3 and SMAD4, SMAD1 signaling is likely to be affected as well, due to nuclear translocation in complex with SMAD4. Therefore, assessing the expression of factors such as FoxOs and FoxG1 in MV4-11 and MM6 cells could provide more information the diverging effects of SMAD1 in the two cell lines.

In conclusion, we showed that the loss of *SMAD1* expression, in AML patient samples and cell lines with *KMT2A::AFF1* and *KMT2A::MLLT3*, might contribute to leukemogenesis by influencing the response to TGF-β and the individual downstream effects of it.

Future studies could evaluate whether the pharmacological induction of *SMAD1* expression may represent a therapeutic strategy in *KMT2A*-rearranged AML with *KMT2A::AFF1* and *KMT2A::MLLT3* fusion genes.

## Data Availability

The original contributions presented in the study are included in the article/**Supplementary Material**. Further inquiries can be directed to the corresponding author.
