# Supplementary figures and images for: Loss of SMAD1 in acute myeloid leukemia with KMT2A::AFF1 and KMT2A::MLLT3 fusion genes
Source: Front Oncol. 2025 Jan 6;14:1481713. doi: 10.3389/fonc.2024.1481713 (PMC11743462; doi:10.3389/fonc.2024.1481713)

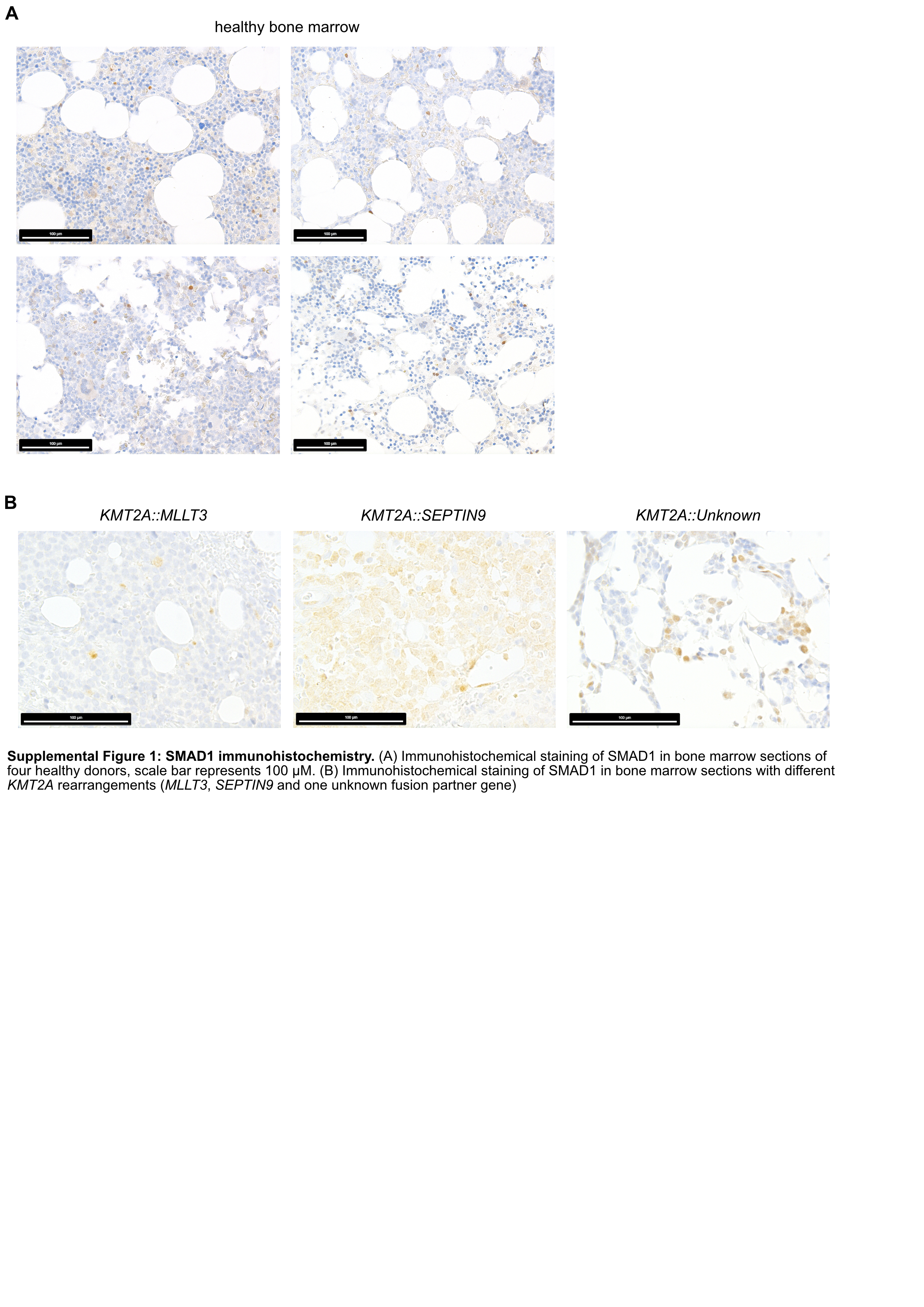

Supplement: Supplementary file 1 [file Image1.jpeg]

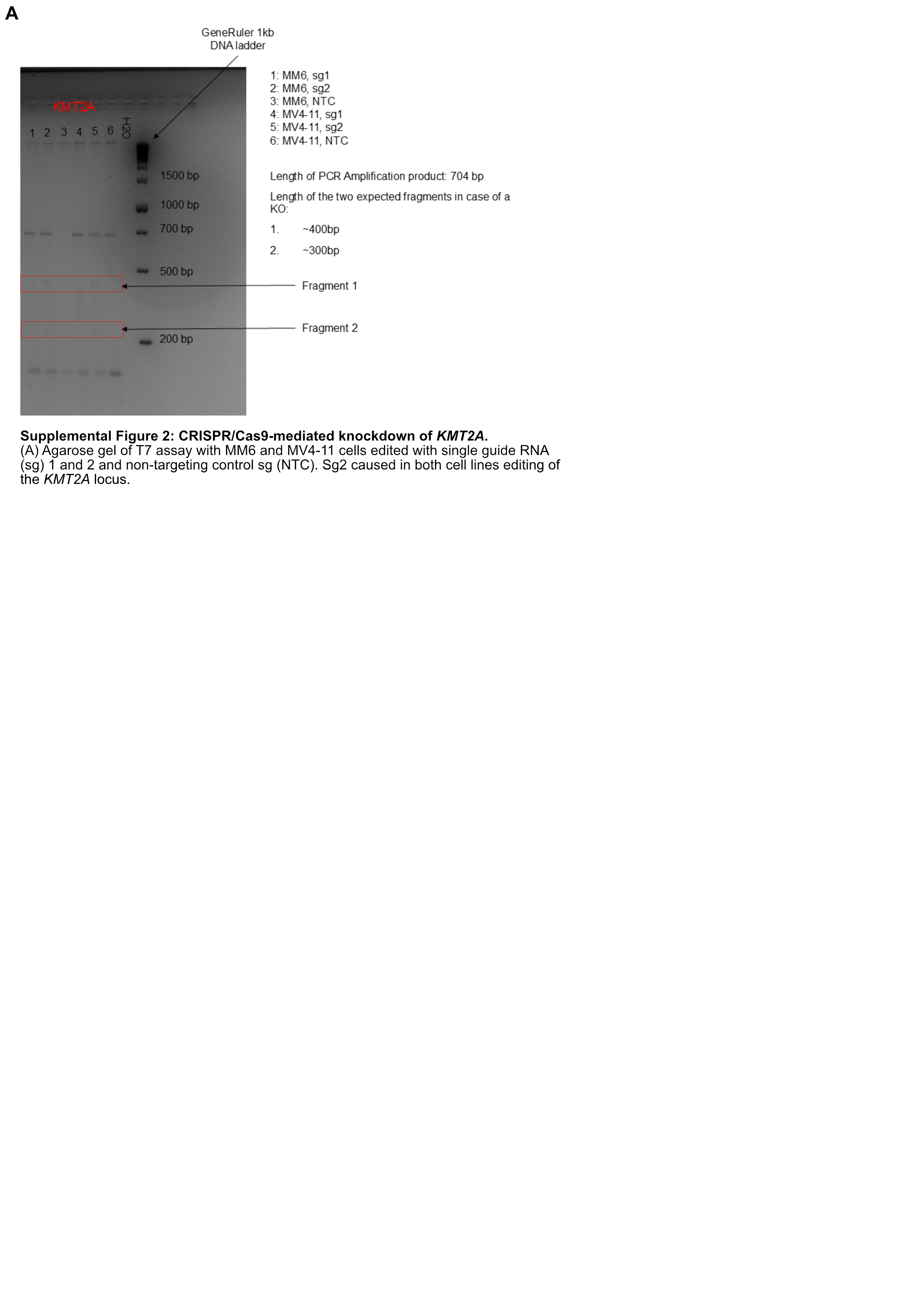

Supplement: Supplementary file 2 [file Image2.jpeg]

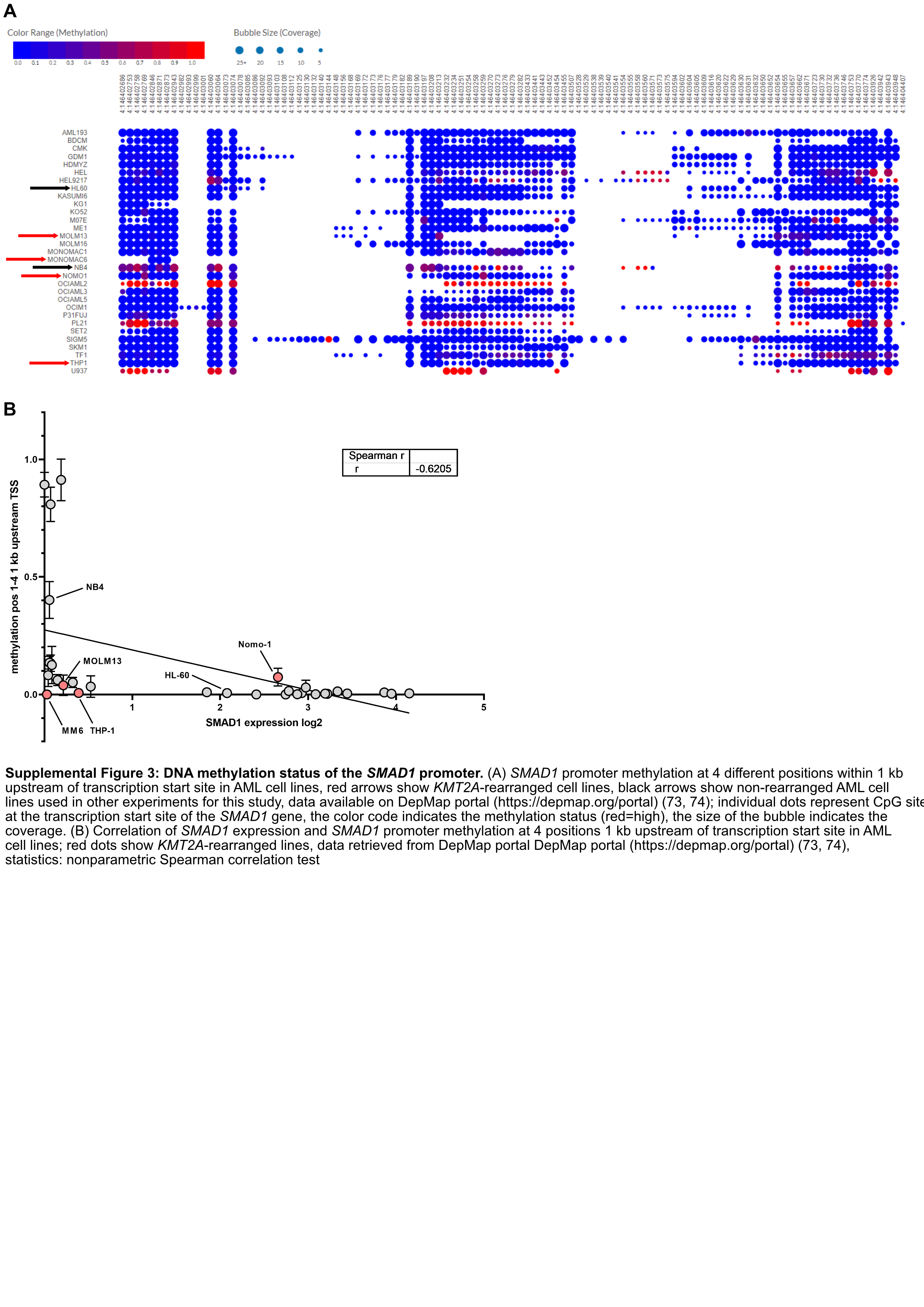

Supplement: Supplementary file 3 [file Image3.jpg]

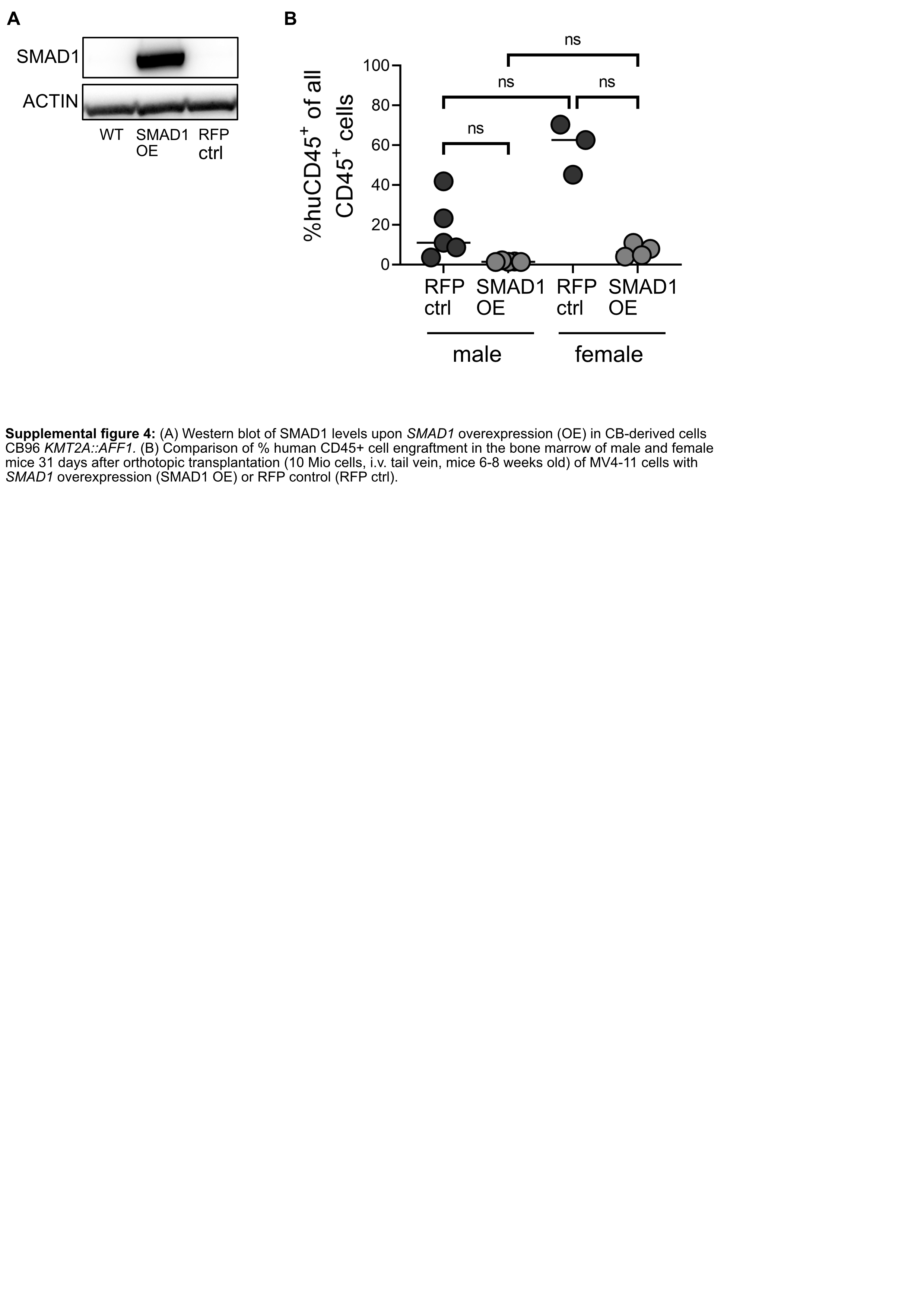

Supplement: Supplementary file 4 [file Image4.jpg]
