# Supplementary material for: Loss of SMAD1 in acute myeloid leukemia with KMT2A::AFF1 and KMT2A::MLLT3 fusion genes
Source: Front Oncol. 2025 Jan 6;14:1481713. doi: 10.3389/fonc.2024.1481713 (PMC11743462; doi:10.3389/fonc.2024.1481713)
Supplement: Supplementary file 5 [file Table1.docx]

**Cell lines and cord blood-derived cells**

| **cell line** | **type** | **gender** | **MLL fusion gene** | **other mutations** |
| --- | --- | --- | --- | --- |
| MV4-11 | acute monocytic leukemia | male | MLL::AF4 | FLT3-ITD |
| MM6 (MONOMAC 6) | acute monocytic leukemia | male | MLL::AF9 | RUNX1::ATP8A2, TP53 |
| MOLM-13 | acute myeloid leukemia | male | MLL::AF9 | FLT3-ITD |
| THP-1 | acute monocytic leukemia | male | MLL::AF9 | CSNK2A1::DDX39B, NRAS, TP53 |
| Nomo-1 | acute monocytic leukemia | female | MLL::AF9 | EP300, KRAS, TP53 |
| RS4-11 | B acute lymphoblastic leukemia | female | MLL::AF4 |  |
| HL-60 | acute myeloid leukemia | female |  | TP53, CDKN2A, NRAS |
| CMK115 | acute megakaryoblastic leukemia | male |  | trisomy 21, TP53::FXR2, TP53 |
| Marimo | therapy-related acute myeloid leukemia | female |  | CALR, MPL, NRAS, TP53 |
| NB4 | acute promyelocytic leukemia | female |  | PML::RARA, KRAS, TP53 |
| ENL | Cord blood Lin- cells, pro-B-cell-like |  | MLL::ENL (transgenic) |  |
| AF92 | Cord blood Lin- cells, pro-B-cell-like |  | MLL::AF9 (transgenic) |  |
| AF93 | Cord blood Lin- cells, biphenotypic (CD19+, CD33+) |  | MLL::AF9 (transgenic) |  |
| CB63 | Cord blood CD34+ |  | MLL::AF4 Intron 11 breakpoint |  |
| CB96 | Cord blood CD34+ |  | MLL::AF4 Intron 11 breakpoint |  |
| CB65 | Cord blood CD34+ |  | MLL::AF9 Intron 11 breakpoint |  |
| CB54 | Cord blood CD34+ |  | MLL::AF9 Intron 11 breakpoint |  |
| CB126 | Cord blood CD34+ |  | MLL::AF9 Intron 9 breakpoint |  |

**Supplemental table 1.:** Cell lines and cord blood-derived cells.
