# Supplementary material for: Loss of SMAD1 in acute myeloid leukemia with KMT2A::AFF1 and KMT2A::MLLT3 fusion genes
Source: Front Oncol. 2025 Jan 6;14:1481713. doi: 10.3389/fonc.2024.1481713 (PMC11743462; doi:10.3389/fonc.2024.1481713)
Supplement: Supplementary file 6 [file Table2.docx]

**RT-qPCR and ChIP-qPCR primers**

| **method** | **target** | **forward** | **reverse** | **species** |
| --- | --- | --- | --- | --- |
| RT-qPCR | *SMAD1* | ACC TGC TTA CCT GCC TCC TG | CAT AAG CAA CCG CCT GAA CA | human |
| RT-qPCR | *MEIS1* | AAGCAGTTGGCACAAGACACGG | CTGCTCGGTTGGACTGGTCTAT | human |
| RT-qPCR | *HOXA9* | AGAATGAGAGCGGCGGAGACAA | CTCTTTCTCCAGTTCCAGGGTC | human |
| RT-qPCR | *MLL1-N* | TCAGCTGCAGGGAAGAAAGG | CTATAAACCGCCGAGGGGTC | human |
| RT-qPCR | *MLL1-C* | GGCCTGAATTTCTCCACAGA | TTCGACAGACGCTGTAGGTG | human |
| RT-qPCR | *MLL::AF4* | CACCTACTACAGGACCGCCAA | GGGGTTTGTTCACTGTCACTGTCC | human |
| RT-qPCR | *MLL::AF9* | AACCACCTCCGGTCAATAAGC | TTCACGATCTGCTGCAGAATG | human |
| RT-qPCR | *GAPDH* | ACAACAGCCTCAAGATCA | TTCTGGGTGGCAGTGATG | human |
| RT-qPCR | *ACTIN* | CACCATTGGCAATGAGCGGTTC | AGGTCTTTGCGGATGTCCACGT | human |
| RT-qPCR | *RPLP32* | GAA GTT CCT GGT CCA CAA CG | GCG ATC TCG GCA CAG TAA G | human |
| ChIP-qPCR | *SMAD1* promoter region | CTT TCC CTT TAC CGG AGT CG | CAA GCC CAC ATC TGT CTC TC | human |
| ChIP-qPCR | *MYOD1* promoter region | CGG GGC GTT TAG GCT ACT AC | CTC CTG TCC GGC CTG ATT TG | human |
| ChIP-qPCR | *EIF4A2* promoter region | GGG GAA AGC GAG GTT TAA CT | TTA CAG GGT CGC TGG AAA | human |

**Supplemental table 2.:** RT-qPCR and ChIP-qPCR primers.
