# Supplementary material for: Loss of SMAD1 in acute myeloid leukemia with KMT2A::AFF1 and KMT2A::MLLT3 fusion genes
Source: Front Oncol. 2025 Jan 6;14:1481713. doi: 10.3389/fonc.2024.1481713 (PMC11743462; doi:10.3389/fonc.2024.1481713)
Supplement: Supplementary file 7 [file Table3.docx]

**Western Blot and ChIP-qPCR antibodies**

| **method** | **target** | **company** | **catalogue #** |
| --- | --- | --- | --- |
| Western Blot | SMAD1 | Cell Signaling Technology | 9743S |
| Western Blot | ACTIN | Merck Millipore | MAB1501R Clone 4 |
| Western Blot | GAPDH | Thermo Fisher Scientific | MA5-15738 (GA1R) |
| Western Blot | TUBULIN | Sigma-Aldrich | 05-829 (DM1A) |
| ChIP-qPCR | H3K4me3 | Diagenode | C15410030 |
| ChIP-qPCR | IgG | Diagenode | C1510206 |

**Supplemental table 3.:** Western Blot and ChIP-qPCR antibodies.
